# Pulmonary Hypertension as a Primary Presentation for Schistosomiasis, a Case Report of Unusual Presentation

**DOI:** 10.1093/omcr/omz073

**Published:** 2019-08-10

**Authors:** Mogahed Ismail Hassan Hussein

**Affiliations:** Department of Internal Medicine, Faculty of Medicine, University of Gezira, Wad Medani City, Sudan

## Abstract

The combination of schistosomiasis and pulmonary hypertension (PH) was always recognized as a very rare one; in medical literature, PH is considered as a manifestation of hepatosplenic schistosomiasis but not a manifestation of schistosomal infection until recently. Only 18.5% of patients that have a documented hepatosplenic schistosomiasis were found with PH. Schistosomiasis rarely causes PH without evident hepatosplenic manifestations. Here, we are reporting a case of a patient whose first clinical presentation was features of PH. We use this case as an opportunity to outline pathological mechanisms, causes and classification of PH. A structured and thorough workup for PH is emphasized. It is important to exclude all other secondary causes to be able to diagnose primary PH especially in the absence of a positive family history and advanced diagnostic technology.

## INTRODUCTION

**Figure 1:**
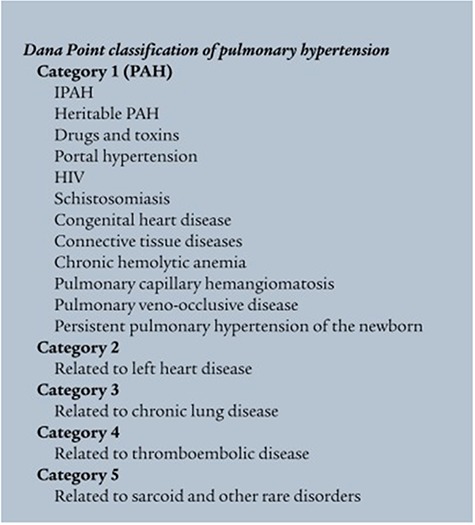
Dana Point 2008, classification.

Pulmonary hypertension (PH) is considered when the mean pulmonary artery pressure at rest is equal or more than 25 mm Hg [1]. The fourth world symposium on PH took place in Dana Point 2008 and provided slight modifications to the classification scheme [2]. This categorization is detailed further in our discussion (Fig. 1). In this report, we describe the rare case of a patient who was presented with PH secondary to schistosomal infection without any identifiable symptoms or signs of hepatosplenic disease.

## CASE REPORT

A 26-year-old farmer was presented to the internal medicine department with the complaint of shortness of breath, New York Heart Association class IV. He denied any chest pain, palpitations, stridor, syncope, cough or haemoptysis. The patient denies weight loss or systemic features like fevers or malaise. The patient denies abdominal distension, hematemesis, skin changes and dysphagia. He is sexually active with his wife, no past medical history of syphilis.

Clinical examination of the cardiovascular and palpation of the precordium shows a palpable P2 and positive left parasternal heave. Electrocardiogram performed in clinic showed a significant right axis deviation. Abdominal and hand examinations were normal. Given these clinical findings, it was felt that a PH was the most likely cause of his symptoms.

Echocardiogram (ECHO) was thereafter performed to estimate the pulmonary artery systolic pressure, which was 28 mm Hg, no right ventricle dysfunction or any finding that would suggest a cardiac cause.

**Figure 2:**
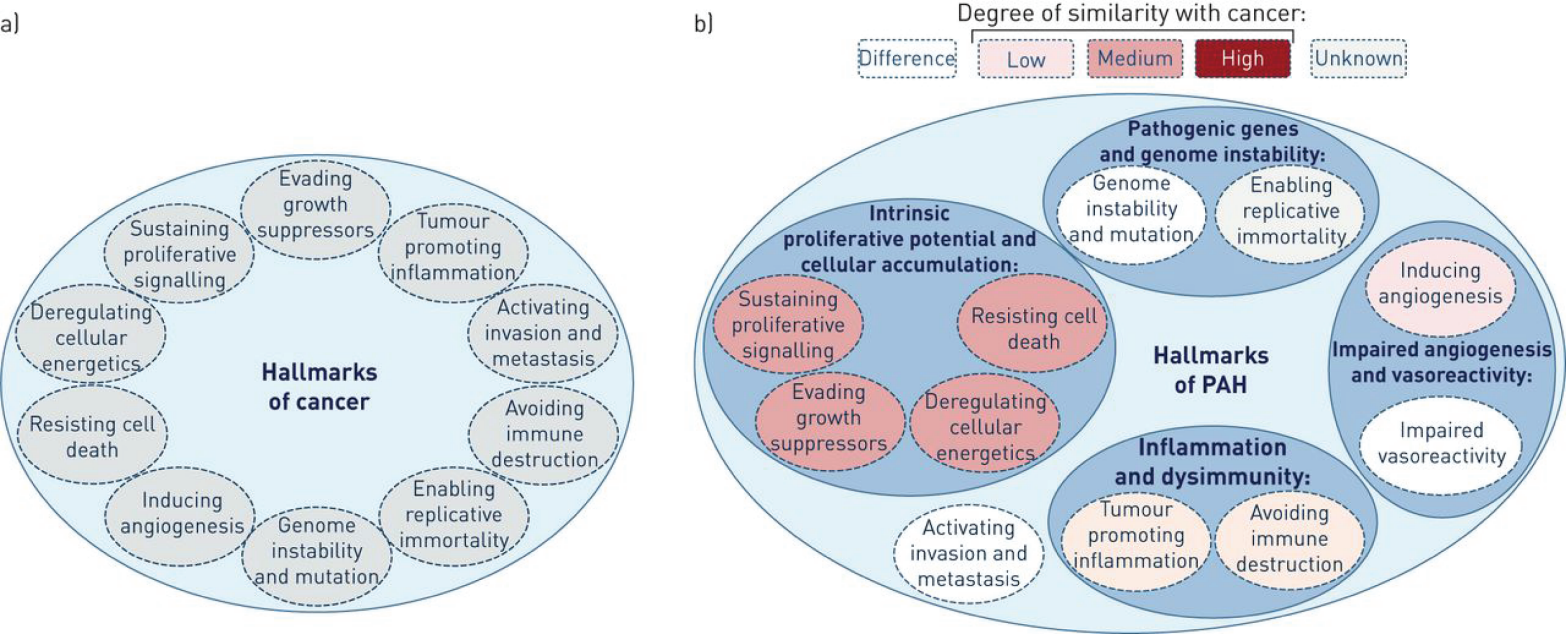
(**A**) In 2000 scientists have proposed six capabilities that are similar to cancer molecular conditions, and they added four additional hallmarks in 2011. (**B**) PAH pathophysiology resembles cancer to a variable degree. No invasion or metastasis was observed in PAH. In addition, these features found in PAH also appear to contribute to the disease pathogenesis with different levels of importance. This was reproduced with permission from the *European Respiratory Journal*.

The patient was concerned about his problem, he received sildenafil tabs and his condition improved. He was also given warfarin as several studies, using both univariate and multivariate analyses, have shown that survival in idiopathic pulmonary arterial hypertension (IPAH), regardless of histopathologic subtype, is increased when patients are treated with anticoagulant therapy [3]. Upon further inquiry at the follow-up, the patient revealed that he used to swim and bathe in a local pool. He had a past history of schistosomiasis diagnosed by microscopic egg detection, which is the gold standard for diagnosis of schistosomiasis. It was concluded that the patient is an asymptomatic carrier of schistosomiasis; abdominal ultrasound was done and showed normal spleen and liver along with thin periportal fibrosis. The past history of schistosomal infection along with the periportal fibrosis, negative syphilis screening and normal heart structure makes schistosomal PH is the likely diagnosis.

## DISCUSSION

There is no single pathological mechanisms for PH; instead, many different mechanisms are implicated in the pathogenesis of IPAH, a combination of pulmonary vasoconstriction, *in situ* thrombosis and pulmonary arterial wall remodelling, which are responsible for the rise in pulmonary vascular resistance (PVR) and pulmonary arterial pressure in patients with PAH, causing a progressive functional decline in patients despite advancing therapies [4]. Lately, scientists had come to a wonderful understanding and released a novel cancer-like concept for PAH [3, 5] (Fig. 2). Vasoconstriction theory due to the imbalance between locally produced vasodilators such as nitric oxide and prostacyclin and vasoconstrictors such as endothelin and thromboxane is a major contributor to the observed increase in the PVR, and it is considered one of the pathobiologic basis of therapy. The manifestations of the disease are not specific, hence why misdiagnosis may occur. The presence of PH is clinically suggested by the presence of dyspnea in the absence of heart or respiratory disease. The physical include left parasternal heave, a loud pulmonary component of the second heart sound (P2), a pan systolic murmur of tricuspid regurgitation, a diastolic murmur of pulmonary insufficiency and right ventricular S3. Raised jugular venous pressure, palpable liver, lower limb oedema, ascites causing abdominal distention and cold extremities are all features of advanced disease. Central cyanosis might also be present.

**Figure 3:**
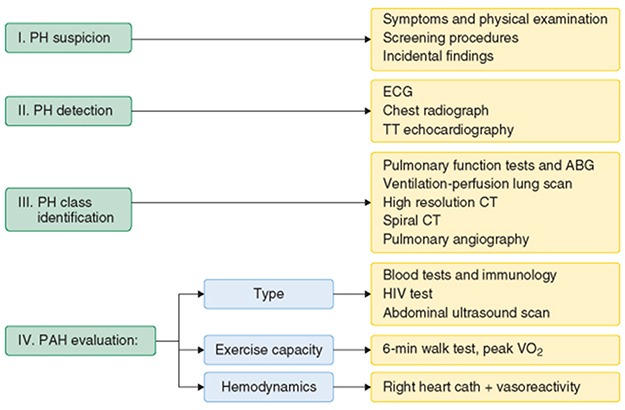
Simplified diagnostic approach to pulmonary hypertension. ABG means arterial blood gases; cath, catheterization; CT, computed tomography; ECG, electrocardiogram; HIV, human immunodeficiency virus; PH, pulmonary hypertension; PAH, pulmonary arterial hypertension; TT, transthoracic; VO2, oxygen consumption (6). This was reproduced from Cleveland clinic center for continuing education, and it is no longer subject to copyrights.

Structured algorisms have been released to guide health practitioners to provide more accurate and detailed work up for the disease (Fig. 3) [6].

There are three different pathophysiologic mechanisms behind PH that is caused by schistosomiasis. Schistosomal egg migration to pulmonary arteries was the first mechanism to be described. It occurs in case of periportal fibrosis due to parasite-induced granulomas, and it usually not associated with cirrhosis. The egg will cause pulmonary arterial obstruction, which will cause a plexiform lesion [7]. Wide and asymmetric vasculopathy is the second proposed mechanism and it was named obliterative arteritis. It may be caused by autoimmune mechanism or associated with egg burden [8]. The final theory proposed that the schistosomal PH is a variant of porto-PH. As the pulmonary arteries have a high flow state, this theory first hypothesised the development of hepatosplenic disease, then portal hypertension and finally PH, and this explains the cases of PH in the setting of chronic hepatosplenic disease with no evidence of granulomas or egg migration to the pulmonary arteries.

In conclusion, schistosomiasis is a very common parasitic infection that has a wide range of complications like systemic anaphylaxis reaction, hepatic, pulmonary, and CNS granulomatous involvement. In endemic areas, the disease may have unusual presentations; like in this case, the patient developed PH due to schistosomiasis without symptomatic hepatosplenic disease.

From this we can conclude that in endemic areas, if a patient presents with PH in the background of exposure to the disease risk factors, the physician must maintain a low threshold for abdominal ultrasound to check for periportal fibrosis and for doing other disease-specific investigations.
